# A hypothesized TNM staging system based on the number and location of positive lymph nodes may better reflect the prognosis for patients with NSCLC

**DOI:** 10.1186/s12885-019-5797-8

**Published:** 2019-06-17

**Authors:** Xiaoling Shang, Jia Liu, Zhenxiang Li, Jiamao Lin, Haiyong Wang

**Affiliations:** 1 Department of Clinical Laboratory, Qilu Medical College, Shandong University, Jinan, 250117 China; 2 Department of Respiration, Qianfo Shan Hospital of Shandong, Jinan, Shandong 250021 People’s Republic of China; 3 Department of Radiotherapy, Shandong Cancer Hospital and Institute, Shandong First Medical University and Shandong Academy of Medical Sciences, Jinan, 250117 China; 4 Department of Internal Medicine-Oncology, Shandong Cancer Hospital and Institute, Shandong First Medical University and Shandong Academy of Medical Sciences, Jinan, 250117 China

## Abstract

**Background:**

This study aimed to evaluate the feasibility and prognostic accuracy of incorporating the number of positive lymph nodes (PLN) into the TNM staging system for non-small cell lung cancer (NSCLC) patients.

**Methods:**

We screened a total of 9539 patients with resected stage IA-IIIB non-small cell cancer between 2010 and 2015 from SEER database. The chi-square test was used to compare patient baseline characteristics and the X-tile model was applied to determine cut-off values for the number of PLN (nN). The X-tile model was used to screen three different cut-off values including nN = 0, nN1–3 and nN4-. Univariate and multivariate Cox proportional hazards regression models were used to analyze the influence of different variables on overall survival (OS). Kaplan-Meier and log-rank test were used to compare survival differences.

**Results:**

Based on the nN cutoffs, we conducted the univariate and multivariate Cox proportional hazards regression. The result showed that nN stage was a significant prognostic factor affecting patients' OS (all *P* <  0.001). We reclassified the seventh edition TNM stages of the enrolled patients with stage IA-IIIB NSCLC according to the 5-year OS rate. Hypothesized TNM substage based on the location and the number of PLN was further calculated. Then we drew survival curves for each substage, including for the current TNM stage and the hypothesized TNM stage. From the comparison of survival curves, we found that the survival curve of each substage of the hypothesized TNM classification was proportional and well distributed compared with the current TNM classification (*P* <  0.001).

**Conclusion:**

Revised TNM staging integrating locational pN stage and numerical nN stage was a more accurate prognostic determinant in patients with NSCLC.

## Background

Lung cancer is the leading cause of malignancy-related deaths in males and is second among female cancer patients worldwide [1, 2]. Non-small cell cancer (NSCLC) makes up approximately 85% of all lung cancers [3, 4]. The tumor-node-metastasis (TNM) staging system is an essential prognostic assessment tool for decision-making about the most appropriate stage-specific therapeutic strategy for NSCLC patients [5].

An accurate assessment of lymph node involvement is essential for the diagnosis and treatment of NSCLC, including the location of the metastatic lymph nodes and the number of positive lymph nodes. Some studies [6, 7] have demonstrated that the number of positive lymph nodes is an important prognostic factor for resected NSCLC. Nwogu CE et al. [8] demonstrated that both the resection of more lymph nodes (LNs) and low ratios of positive LNs to total examined LNs are associated with superior patient survival after NSCLC resection independent of age, sex, grade, tumor size and disease stage. Wei S et al. [9] demonstrated that the nN category was a better prognostic determinant than location-based pN stage classification.

The number of positive lymph nodes is considered as part of the TNM staging system in breast, gastric, and colorectal cancer [10, 11]. However, the seventh edition [12] and the revised eighth edition [13] TNM staging system defined nodal status depending only on the location of the metastatic lymph nodes and does not involve the number of positive lymph nodes. To the best of our knowledge, the number of positive lymph nodes has not been tested as the one criterion of TNM staging.

Accurate staging at the time of initial diagnosis is very important for patients with NSCLC to determine prognosis and guide treatment [14, 15]. The current seventh edition TNM staging system was published in 2009^12^ and the revised eighth edition TNM staging system was published in 2015 [13]. TNM stages include three components: primary tumor (T), nodal status for metastasis (N), and metastasis at the distant organs (M). The eighth TNM staging system included notable changes in the T and M descriptors and in nodal map, while the N descriptor remained the same as in the previous version.

Nodal status is a significant factor in staging, including the location of the metastatic LNs and the number of metastatic LNs, and should predict survival of NCSLC patients after surgery. The nodal status of the current TNM staging assesses tumor burden in the regional hilar and mediastinal nodes [16] and is defined as N0 (no nodal involvement), N1 (peribronchial, interlobar, hilar node involvement), N2 (ipsilateral nodal involvement), N3 (contralateral mediastinal, contralateral hilar or supraclavicular nodal involvement), depending on the location of the metastatic lymph nodes. The number of PLNs has been shown to be a prognostic factor for resected NSCLC [9, 17–19]. An accurate PLN metastasis assessment is crucial in determining treatment, as the prognosis of lung cancer is directly proportional to the PLN.

Saji H et al. [17] demonstrated that resection of ≥10 LNs influenced survival and that the number of involved LNs (four and more) was a strong independent prognostic factor in NSCLC. In another relevant study [18], they showed that a combined anatomically based pN stage classification and numerically based nN stage classification was a more accurate prognostic determinant in patients with NSCLC, especially in the prognostically heterogeneous pN1 and pN2 cases. Similarly, other research [19–22] also showed that the number of lymph nodes and lymph node ratio were important in TNM classification for NSCLC. Furthermore, Asamura H and his colleagues [23] recommended that physicians recorded the number of metastatic lymph nodes (or stations) to further classify N category using new descriptors, such as N1a, N1b, N2a, N2b, and N3, for further testing in the other study.

In NSCLC, similar to colorectal, breast and bladder cancer [24–26], the number of positive lymph nodes has been proven to be a prognostic factor and impacts disease survival [21, 27, 28]. In solid tumors, the number of metastatic lymph nodes has been included in the TNM staging system, such as breast, gastric, and colorectal tumors. However, current pN staging of NSCLC depends only on the location of the metastatic lymph nodes.

In this study, we retrospectively evaluated the association between the number of PLN and the prognosis of patients with resected stage IA-IIIB NSCLC and compared this hypothesized TNM staging system with the current TNM stage classification in terms of prognostic accuracy.

## Methods

### Data source

The SEER Program (www.seer.cancer.gov), a total of 18 population-based cancer registries in the United States (USA), is published annually by the Data Analysis and Interpretation Branch of the National Cancer Institute, MD, USA [29]. The SEER*Stat software (SEER*Stat 8.3.5) was used to identify appropriate patient data. Using this software and according to American Joint Committee on Cancer (AJCC) seventh edition TNM stage, we screened a total of 9539 patients with resected stage IA-IIIB NSCLC between 2010 and 2015. The inclusion criteria was as follows: patients diagnosed IA-IIIB NSCLC and had surgical resection without distant metastasis as well as only one primary tumor and active follow-up. Patients with stage IV NSCLC, incomplete resection, unknown TNM stage, unknown clinical information and benign tumor were excluded. In addition, patients with missing values for positive lymph nodes counts and clinical features were also excluded.

### Ethics statement

This study was mainly based on the SEER database and was conducted in compliance with the Helsinki Declaration. The informed consent was not required because personal identifying information was not involved. This study was approved by the ethics committee of the Shandong Cancer Hospital and Institute.

### Statistical analysis

For all patients, the following variables were analyzed: age, race, sex, histology, pT stage, pN stage and No. of PLN. OS was regarded as the primary endpoint in this study. Other endpoints were the 1-year, 2-year, 5-year OS rate, especially 5-year OS rate. Differences of patient baseline characteristics were analyzed using the chi-square test. Kaplan-Meier analysis was used to draw the survival curves. Differences in survival were examined using the log-rank test. Additionally, the X-tile model was applied to determine the cut-off values of the number of PLN. Univariate and multivariate Cox proportional hazards regression models were used to evaluate the prognostic factors on OS for NSCLC patients. All statistical analyses were made using Statistical Product and Service Solutions (SPSS) 22.0 software package. All statistical *P* values were 2-sided and *P* <  0.05 was considered statistically significant.

## Results

### Patients characteristics

A total of 9539 patients with resected stage IA-IIIB NSCLC from the SEER database were included in our analysis. Most patients were diagnosed at older than 65-year-old (62.5%) and belonged to white race (84.5%). Adenocarcinoma was the main pathology (63.2% vs. 36.8%). According to AJCC stage, patients with T1, 2, 3, 4 stage tumors were 36.2, 43.5, 15.7 and 4.6%, respectively. In addition, patients with pN0, 1, 2, 3 stage were 65.5, 19.4, 14.9 and 0.2%, respectively. Overall, 66.9% patients had no positive LNs and 33.1% patients had more than one PLN. The baseline characteristics of patients were listed in Table 1.

**Table 1:** Resected pathological staged IA-IIIB non-small cell lung cancer (NSCLC) patient characteristics from SEER Database

	Number	%
Total	9539	100
Age		
< 65	3579	37.5
≥ 65	5960	62.5
Race		
White	8063	84.5
Black	780	8.2
Others	696	7.3
Sex		
Female	4643	48.7
Male	4896	51.3
Histology		
Adenocarcinoma	6033	63.2
Squamous	3506	36.8
pT stage		
T1	3454	36.2
T2	4146	43.5
T3	1497	15.7
T4	442	4.6
pN stage		
pN0	6249	65.5
pN1	1852	19.4
pN2	1418	14.9
pN3	20	0.2
No. of PLN		
0	6383	66.9
≥ 1	3156	33.1

### Current TNM staging and survival

**Fig. 1:**
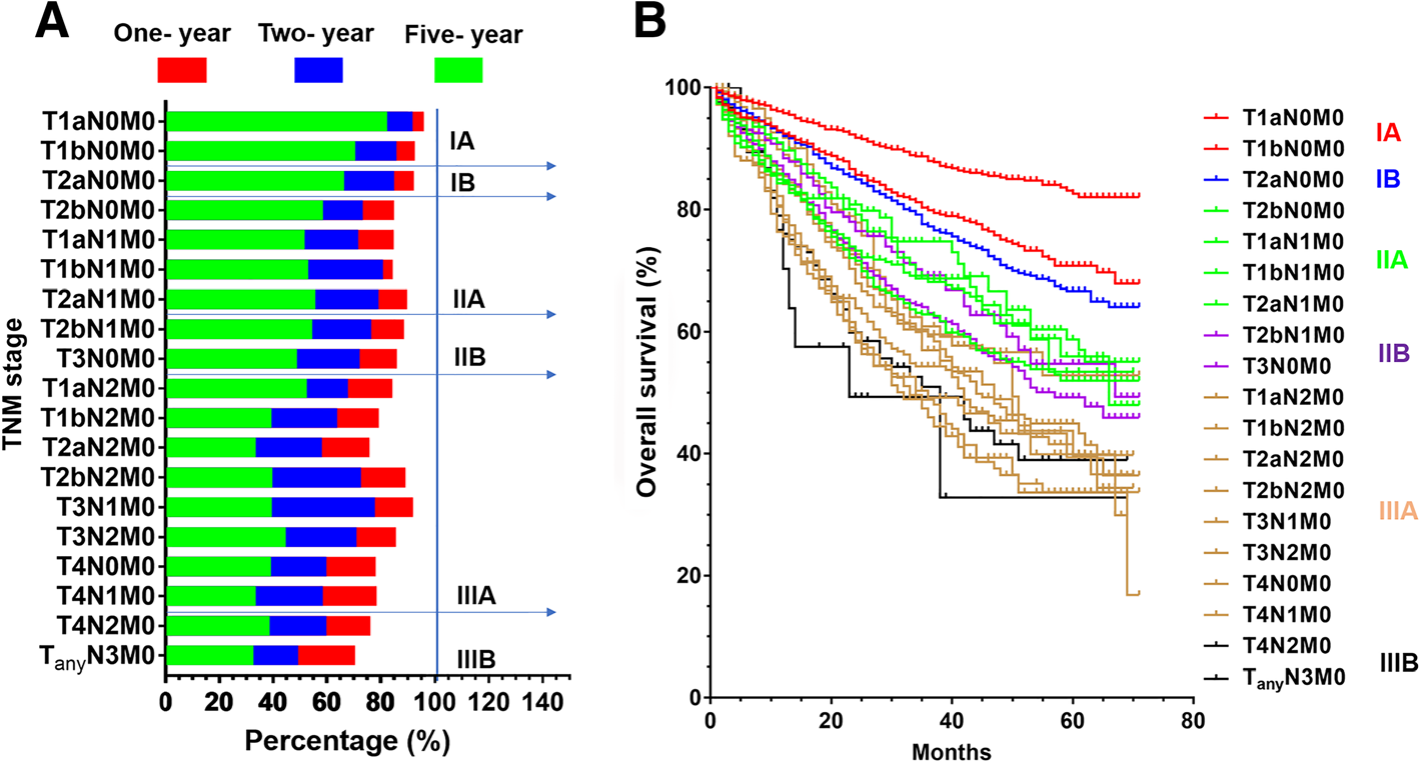
**a**: Comparison between 1-, 2-, 5-year OS rate in different substage using current TNM staging. **b**: Comparison between survival curves for each substage using the current TNM staging system

Stages were classified according to the seventh TNM staging system based on clinical stage. Survival curves were measured using the Kaplan-Meier and log-rank test to compare survival differences between different stages. Survival analysis was performed according to different T category, N category, and stage group in each substage. The 1-, 2-, 5-year survival of different substages of the current TNM stage were compared. It was found that the 5-year OS rate of T2bN1M0 in stage IIB was better than T1aN1M0 in stage IIA; T1aN2M0 in stage IIIA was better than T3N0M0 in stage IIB; T4N2M0 in stage IIIB was better than T2aN2M0 as well as T4N1M0 in stage IIIA.The specific information about 1-, 2-, 5-year OS rate of the current TNM substages is shown in Fig. 1a.

Finally, survival curves were drawn for each substage of the current TNM stage. We found that partial survival curve of stage IIB was better than that of stage IIA and the survival of stage IIIB was better than that of stage IIIA. Each substage survival curve was shown in Fig. 1b.

So, improvements to the TNM staging system from the view of survival prognosis should be considered.

### Cut-off determination for No. of PLN and survival

**Fig. 2:**
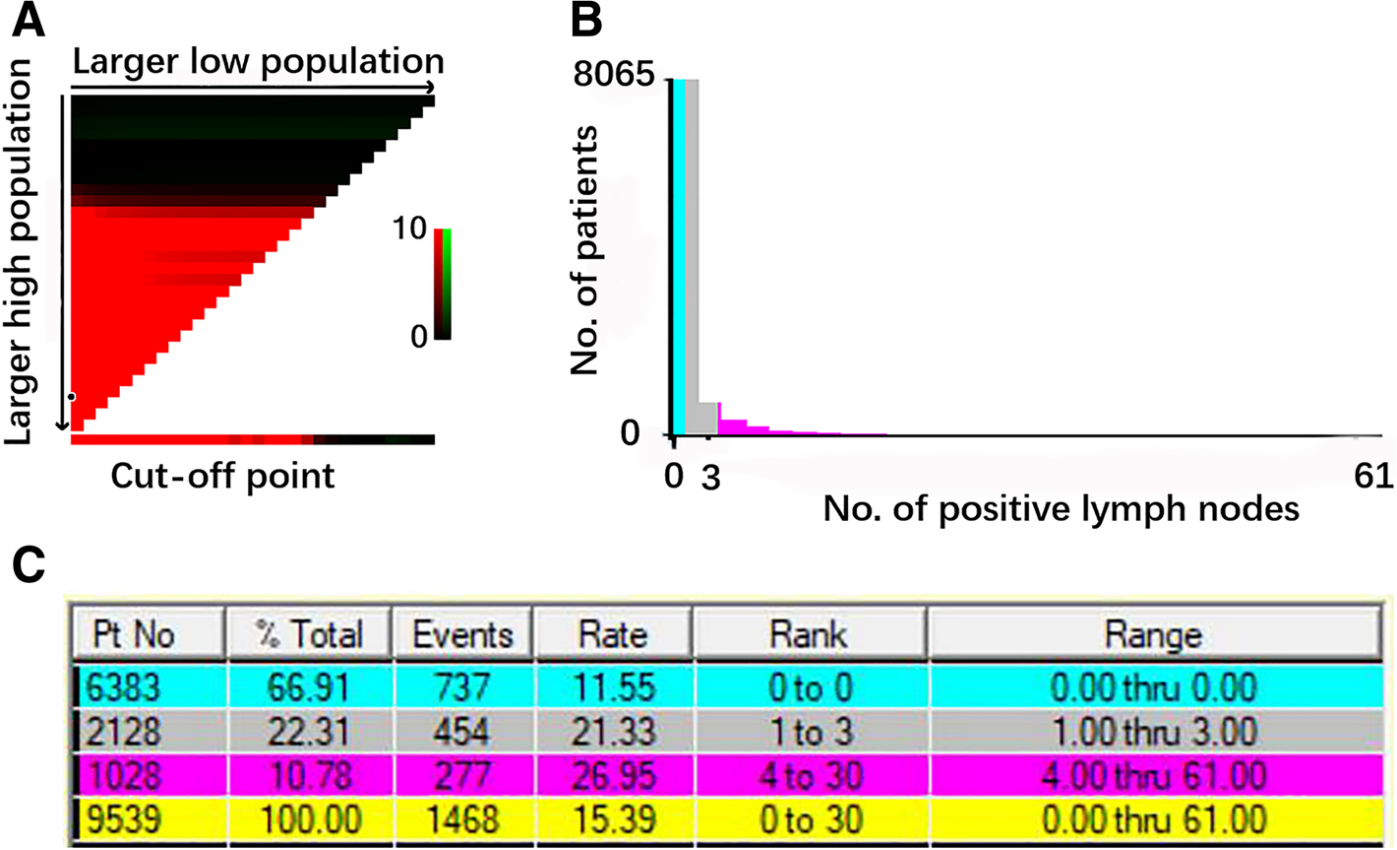
Optimal threshold of No. of PLN count for OS as determined by the X-tile model. **a**: X-tile plots based on No. of PLN. **b**: Optimal cut-off point is shown in the blue (No. of PLN = 0), gray (1 ≤ No. of PLN ≤ 3) and violet panel(No. of PLN ≥ 4) for each PLN cutoff respectively. **c**: specific information about patients with stage nN, blue: 6383 patients (66.91%) with nN0; gray:2128 patients (22.31%) with nN1–3; violet panel: 1028 patients (10.78%) with nN4–61; yellow: a total of 9539 patients

The cut-off values of nN were determined by the X-tile model. The univariate and multivariate Cox proportional hazards regression model were used to evaluate the prognostic value of baseline characteristics. Using the X-tile model, patients were classified into three nN categories: nN0, no LN metastasis; nN1–3, metastasis in one to three PLNs; and nN4-, metastasis in four or more LNs (Fig. 2a, b). Six thousand three hundred eighty-three patients (66.91%) were stage nN0, 2128 patients (22.31%) were stage nN1–3 and 1028 patients (10.78%) were stage nN4-. More specific information about patients with stage nN was shown in Fig. 2c.

Basing on the cut-off values of nN, univariable and multivariable analysis were completed. The results revealed that age, race, sex, histology, pT stage, nN stage were independent prognostic factors affecting patients' OS (all *P* <  0.05). In multivariable analysis, we found that nN stage was an independent and significant prognostic factor on OS for patients with resected stage IA-IIIB NSCLC (nN1–3 VS. nN0: HR = 1.728; 95%CI: 1.571–1.901; *P* <  0.001; nN4- VS. nN0: HR = 2.440; 95% CI: 2.184–2.727; *P* <  0.001) (Table 2).

**Table 2:** Influence of different variables on overall survival (OS) for patients with resected pathological staged IA-IIIB NSCLC analyzed by Cox proportional hazard model

Variables	Univariate analysis		Multivariate analysis	
	Wald χ2	*P*	HR (95% CI)	*P*
Age	75.55	< 0.001		< 0.001
< 65			Reference	
≥ 65			1.468 (1.346–1.601)	< 0.001
Race	6.67	0.036		0.031
White			Reference	
Black			1.004 (0.866–1.165)	0.954
Others			0.799 (0.675–0.945)	0.009
Sex	71.46	< 0.001		< 0.001
Female			Reference	
Male			1.444 (1.326–1.573)	< 0.001
Histology	37.36	< 0.001		< 0.001
Squamous			Reference	
Adenocarcinoma			0.769 (0.707–0.837)	< 0.001
pT stage	143.17	< 0.001		< 0.001
pT1			Reference	
pT2			1.406 (1.267–1.561)	< 0.001
pT3			2.033 (1.800–2.295)	< 0.001
pT4			1.918 (1.607–2.290)	< 0.001
nN stage	286.69	< 0.001		< 0.001
0			Reference	
1–3			1.728 (1.571–1.901)	< 0.001
≥ 4			2.440 (2.184–2.727)	< 0.001

### Current TNM staging system and the hypothesized TNM staging system

**Fig. 3:**
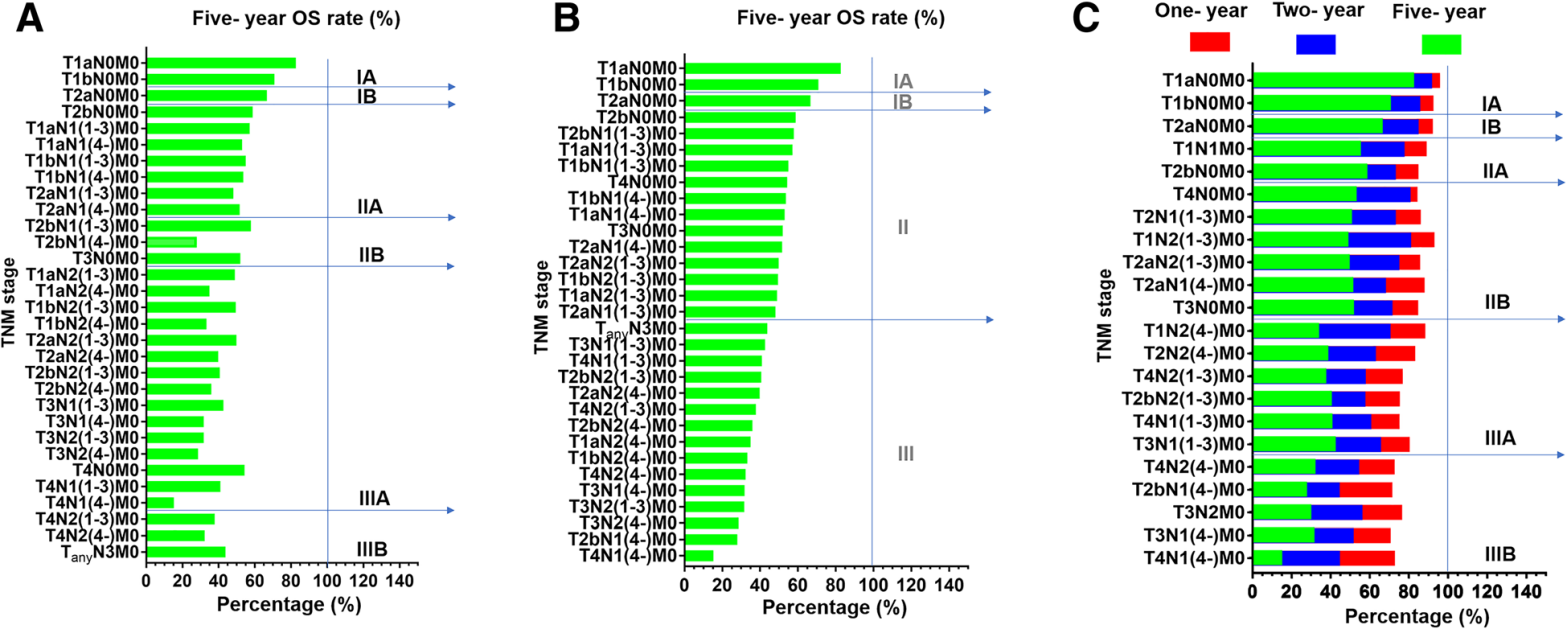
**a**: 5-year OS rate of different substages combined nN stage in current TNM staging system. **b**: 5-year OS rate of different substages combined nN stage according to tendency toward deterioration in hypothesized TNM staging system. **c**: Subclassifications of the hypothesized TNM staging system and the comparison between 1-, 2-, 5-year OS rate of stage IA-IIIB

Then, validation of the nN category in terms of the 5-year OS rate for each pathologic tumor (pT) category was executed (Fig. 3a). According to tendency toward the deterioration of the 5-year OS rate, TNM stage was divided based on the location (pN stage) and the number (nN stage) of positive lymph nodes and obtained the hypothesized TNM stages (Fig. 3b).

The number of stage pN3 cases in the database were too few so we ignored this population. Combined nN stage and pN stage in hypothesized TNM staging, stage II was re-subdivided into IIA, IIB and stage III into IIIA, IIIB. The main changes compared with the classic TNM staging system included reclassification of T2aN1M0 to stage IIB from stage IIA, T1 N2(1–3)M0 and T2aN2(1–3)M0 to stage IIB from IIIA, T4 N2(1–3)M0 to stage IIIA from stage IIIB. Other changes included reclassification of T2bN1(4-)M0 to stage IIIB from stage IIIA and T3 N1(4-)M0, T3N2M0, T4 N1(4-)M0 to stage IIIB from stage IIIA (Fig. 3c).

### Comparison of survival between different TNM staging system

**Fig. 4:**
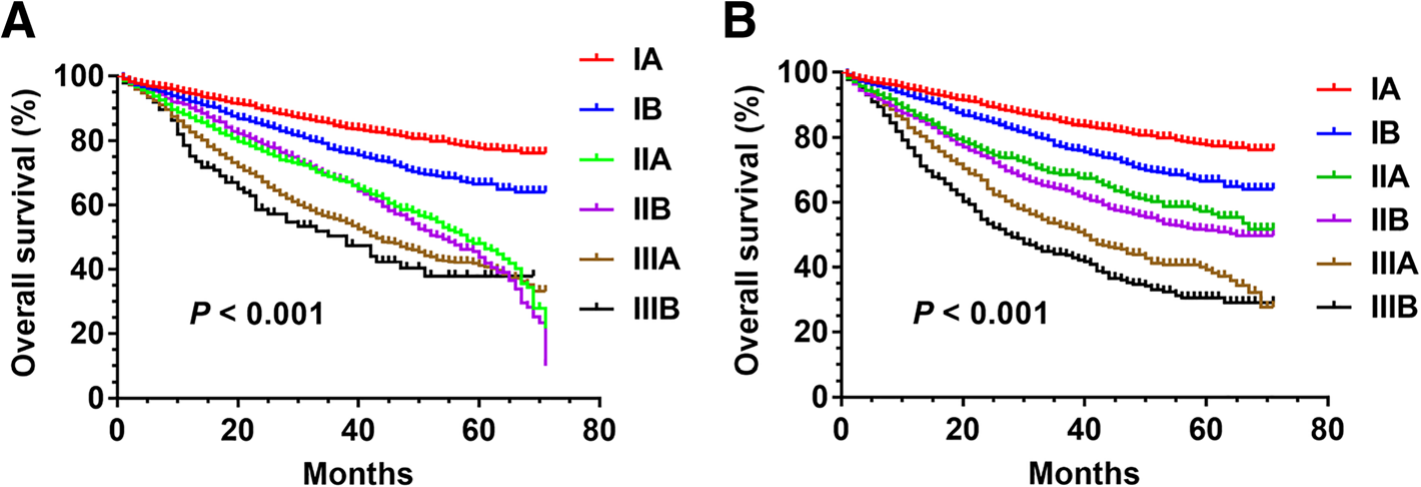
**a**: Survival of patients with resected stage IA - IIIB NSCLC of the current TNM staging system (*P <* 0.001). **b**: Survival of patients with resected stage IA-IIIB NSCLC using the hypothesized TNM staging system (*P <* 0.001)

Survival curves were measured using the Kaplan-Meier and compared by log-rank test. A survival curve was drawn for each substage IA - IIIB with the current TNM classifications (Fig. 4a) and the hypothesized classifications (Fig. 4b), respectively. We found that the hypothesized TNM stages were proportional and well distributed among the survival curves. These results revealed that each survival curve (*P <* 0.001) of the hypothesized subclassification had significant tendency and proportional compared with the survival curve (*P <* 0.001) of the current TNM subclassifications.

## Discussion

In the present study, 1-, 2-, 5-year OS rate were the endpoints. Using the X-tile models, we classified involved the number of PLN into the three nN categories as follows: nN0, no PLN metastasis; nN1–3, metastasis in one to three PLNs; and nN4-, metastasis in four or more PLNs. The outcomes of multivariable analysis had shown that 1 ≤ No. of PLN ≤ 3 and No. of PLN ≥ 4 were independent factors associated with OS (all *P* <  0.001) in patients with stage IA - IIIB NSCLC after surgery. We compared the 1-, 2-, 5-year OS rate for different substages of the current TNM stages, respectively. From this comparison, we found that the current TNM staging system was irrational to guide clinical treatment.

In this study, there were only 20 cases with stage pN3 which accounted for 0.2% of the total patient population. So, we ignored this population and only examined those with stage pN1 and pN2 as the focus of our research. According to nN category and 5-year OS rate, we re-divided the TNM stages and obtained the hypothesized TNM stages. The main changes compared with the current TNM stages included the reclassification of T2aN1M0 to stage IIB from stage IIA, T1 N2(1–3)M0 and T2aN2(1–3)M0 to stage IIB from IIIA, T4 N2(1–3)M0 to stage IIIA from stage IIIB and reclassification of T2bN1(4-)M0 to stage IIIB from stage IIIA and T3 N1(4-)M0, T3N2M0, T4 N1(4-)M0 to stage IIIB from stage IIIA. Survival curves were drawn for both staging system. A comparison showed that the patients with the hypothesized TNM stages had better OS than those with classic TNM staging, although both of *P <* 0.001.

For patients with stage IA-IIIA NSCLC, the first choice treatment is curative tumor resection via surgery [30, 31]. Postoperative chemotherapy and radiotherapy have improved outcomes for these patients and decreased the risk of locoregional recurrence [32–34]. However, Andre F et al. [35] have reported that many patients who have N2+ disease are a heterogeneous group. And the treatment should be individualized for these patients. Surgery alone may be a more limited for patients with stage IIIA-N2 NSCLC [36]. Chemotherapy and radiation are main treatment methods for these patients. In this study, patients with pN2 NSCLC who account for 14.9% were treated with surgery from SEER database. We analyzed the impact of these patients on staging and we did not analyze those patients who did not undergo surgical treatment. This may have an impact on staging system. So, we need to validate this result in future studies.

In addition, Scagliotti GV et al. [37] have reported that patients with stage IIA-IIIB NSCLC could be benifit from neoadjuvant treatment including preoperative chemotherapy, preoperative radiotherapy and targeted treatment. Among stage IIIA-IIIB patients, we screened from the database, neoadjuvant therapy may or may not be accepted. However, due to the limitation of the database, we could not obtain information on neoadjuvant therapy for stage IIIA and IIIB patients and these data may have an impact on clinical prognosis. Even if this information is not available, we could reflect some problems by incorporating the number of lymph nodes into the revised staging compared with conventional staging.

However, this study has any other limitations that should be noted. First, this was a retrospective study. Moreover, it was difficult for physicians to evaluate the number of positive lymph nodes at the time of diagnosed NSCLC. Some other variables, including smoking history, type of surgery, other treatment affecting the prognosis were not included in the analysis. In addition, all data originated from SEER database according to the seventh edition TNM staging system rather than the eighth edition TNM staging system. This may have some influence on the final results. So, the results require further large-scale prospective clinical study to confirm these recommendations.

## Conclusion

This study demonstrated that hypothesized TNM stage based pN stage and nN stage could more accurately reflect survival for patients with NSCLC. If applied, these results may guide clinicians and surgeon in choose more appropriate oncological treatments for NSCLC but further large-scale prospective clinical studies are required to confirm these recommendations.

## Data Availability

The datasets used and analysed during the current study are available from the corresponding author on reasonable request.

## Abbreviations

AJCC: American Joint Committee on Cancer
CI: Confidence interval
HR: Hazard ratio
LNs: Lymph nodes
nN: Number of positive lymph nodes
NSCLC: Non-small cell lung cancer
OS: Overall survival
PLN: Positive lymph nodes
pN: Pathological lymph nodes
pT: Pathological tumor
SEER: Surveillance Epidemiology and End Results
TNM: Tumor-node-metastasis
